# Exploring the Structural Competition between the Black and the Yellow Phase of CsPbI_3_

**DOI:** 10.3390/nano11051282

**Published:** 2021-05-13

**Authors:** Ioannis Deretzis, Corrado Bongiorno, Giovanni Mannino, Emanuele Smecca, Salvatore Sanzaro, Salvatore Valastro, Giuseppe Fisicaro, Antonino La Magna, Alessandra Alberti

**Affiliations:** 1 Istituto per la Microelettronica e Microsistemi, Dipartimento di Scienze Fisiche e Tecnologie della Materia, Consiglio Nazionale delle Ricerche, Z.I. VIII Strada 5, 95121 Catania, Italy; ioannis.deretzis@imm.cnr.it (I.D.); corrado.bongiorno@imm.cnr.it (C.B.); giovanni.mannino@imm.cnr.it (G.M.); emanuele.smecca@imm.cnr.it (E.S.); salvatore.sanzaro@imm.cnr.it (S.S.); salvatore.valastro@imm.cnr.it (S.V.); giuseppe.fisicaro@imm.cnr.it (G.F.); alessandra.alberti@imm.cnr.it (A.A.); 2 Dipartimento di Fisica e Astronomia “Ettore Majorana”, Università di Catania, Via S. Sofia 64, 95123 Catania, Italy

**Keywords:** inorganic lead halide perovskites, CsPbI_3_, density functional theory, high-resolution electron microscopy

## Abstract

The realization of stable inorganic perovskites is crucial to enable low-cost solution-processed photovoltaics. However, the main candidate material, CsPbI_3_, suffers from a spontaneous phase transition at room temperature towards a photo-inactive orthorhombic δ-phase (yellow phase). Here we used theoretical and experimental methods to study the structural and electronic features that determine the stability of the CsPbI_3_ perovskite. We argued that the two physical characteristics that favor the black perovskite phase at low temperatures are the strong spatial confinement in nanocrystalline structures and the level of electron doping in the material. Within this context, we discussed practical procedures for the realization of long-lasting inorganic lead halide perovskites.

## 1. Introduction

Lead halide perovskites are materials that recently emerged as candidates for next generation solar cells, combining high yields with low-cost processing methods. The photovoltaic properties of hybrid halide perovskites were first demonstrated by Miyasaka et al. [1] who showed a photoactive response in methylammonium-based lead iodides and bromides. Since then, ongoing improvements in the composition [2], mixing [3,4], interface engineering [5,6], and architectural optimization [7,8] have resulted in record-high solar cell efficiencies, directly comparable or even superior to present-day silicon-based technology [9]. The realization of these breakthroughs was largely accomplished by the partial or total occupation of the A-site cation in the ABX_3_ perovskite structure by a monovalent organic molecule, like methylammonium (MA) or formamidinium (FA). MAPbI_3_ and FAPbI_3_ perovskite solar cells showed excellent photovoltaic performance [10,11] but suffered from fast degradation under humidity, electromagnetic radiation, or heat [12,13,14]. Therefore, since the very beginning, the presence of the organic component in the perovskite matrix raised questions on the long-term stability of the respective devices. This stability deficit was partially mitigated by compositional mixing (e.g., by forming mixed Cs-FA-MA-Pb-I-Br perovskites), where the more robust lead-bromide bonding [15] and the reduced rotational disorder [16] increased the lifetime of the respective perovskites. However, even in this case, phase segregation (i.e., the separation of the mixed perovskite into elementary non-perovskite phases) [17] remained a critical issue that has yet to be settled.

A different strategy for the long-term stability of lead halide perovskites is the substitution of the organic cation from the ABX_3_ matrix with a monovalent inorganic cation having a large ionic radius, like cesium. The CsPbI_3_ perovskite could ideally be used in both tandem or single-junction solar cells, having a direct bandgap of ~1.73 eV and favorable optoelectronic characteristics [18] (whereas the CsPbBr_3_ perovskite has a bandgap of ~2.35 eV [19], which significantly restricts its application range in optoelectronic devices). The perovskite phase of CsPbI_3_ is however not the most stable phase at room temperature, where a photo-inactive orthorhombic yellow phase prevails (δ-phase) [20]. The phase diagram of the material indicates a spontaneous transformation from this yellow phase to the black α-perovskite structure only above 300 °C, which is impracticable for solar cell applications. In this respect, the main challenge for the utilization of CsPbI_3_ as an active material in light absorbers is the stabilization of the perovskite phase at a temperature range useful for common optoelectronic devices (20–80 °C). This issue has been a subject of investigation in numerous literature studies, where various strategies have been applied to engineer a stable perovskite phase for CsPbI_3_ close to room temperature. Some of the most prominent approaches are regarding the introduction of bivalent cations like Eu^2+^ in the perovskite lattice [21], the stabilization through strain engineering [22], or the enhancement of the process control using HI (or HPbI_3_) [18,23], water [24], or other additives [25] during the perovskite synthesis. Although these appear to be viable routes for the preservation of the perovskite phase at device operating temperatures, understanding the physical properties that regulate the balance between the two competing phases in CsPbI_3_ is still unclear.

In this paper, we use theoretical (density functional theory) and experimental (X-ray diffraction, high-resolution scanning transmission electron microscopy, spectroscopic ellipsometry) techniques to investigate the impact of various physical characteristics in competition between the yellow and the perovskite phase of CsPbI_3_. We find that the two physical parameters that can shift the phase equilibrium towards the perovskite structure are the electron doping of the material and its spatial confinement in nanometer-sized crystals. We additionally discuss practical process recipes to achieve these objectives. The paper is organized as follows: in Section 2 we describe the theoretical and experimental methodology used in this study, in Section 3 we show calculation outcomes and experimental characterizations of our samples, in Section 4 we critically discuss our results, while in Section 5 we provide the conclusions.

## 2. Materials and Methods

CsPbI_3_ films were obtained though solution processing of 1 M of PbI_2_ and 1 M of CsI (Tokyo Chemical Industry Co. Ltd., Tokyo, Japan) in a composite solvent made of DMF and DMSO (3:1 v/v). An EuCl_3_ solution of 0.1 M (Sigma-Aldrich, Darmstadt, Germany) was separately added in a mixed solvent of DMF and DMSO with the same composition as before. After separately stirring the solutions at room temperature for 1 h, 1 mL of PbI_2_/CsI was mixed with 0.5 mL of EuCl_3_ and stirred for a further 1 h. Subsequently, the mixture was deposited on glass substrates and spin-coated in two steps: 1000 rpm for 10 s followed by 5000 rpm for 25 s in ambient environment. The CsPbI_3_ film with Eu/Pb = 5% was then annealed at 80 °C on a hot plate for 20 s and cooled to 30 °C at 0.5–1 °C/s to form the black perovskite phase. The final CsPbI_3_ layer had a thickness of ~80 nm. A complete discussion on the preparation procedure can be found in Ref. [26].

XRD patterns were collected with a D8Discoved diffractometer (Bruker AXS GmbH, Karlsruhe, Germany) equipped with a high precision goniometer (0.0001 Å) and a Cu-Ka source with an instrumental broadening of 0.07°. In situ XRD was performed using an DHS 900 sample holder (Anton Paar GmbH, Graz, Austria), equipped with a PolyEther-Ether-Ketone dome filled with nitrogen at a pressure slightly higher than the atmospheric pressure (+0.3 bar). Spectroscopic ellipsometry was performed with a VASE Ellipsometer (J. A. Woollam Co. Inc., Lincoln, NE, USA). Samples were kept in a closed chamber with an overpressure of N_2_. High-resolution imaging was performed by Scanning Transmission Electron Microscopy (STEM) at an accelerating voltage of 200 kV, using a Cs-probe corrected ARM200 (JEOL Ltd, Akishima, Tokyo, Japan), equipped with a cold field emission gun. 

Density functional theory calculations were performed with the plane-wave Quantum Espresso code [27]. We used the Perdew–Burke–Ernzerhof (PBE) implementation [28] of the generalized gradient approximation for the description of the exchange-correlation functional and scalar relativistic ultrasoft pseudopotentials [29] for the description of the ionic cores. Pb-5d semicore electrons were dynamically treated in the same way as valence electrons. Convergence was achieved with a plane-wave cutoff kinetic energy of 49 Ry and an augmented charge density cutoff of 258 Ry. Calculations were performed on CsPbI_3_ unit cell structures comprised of 20 atoms in either the perovskite or the yellow orthorhombic symmetry. A (6 × 6 × 4) Monkhorst-Pack grid [30] was considered for Brillouin zone sampling of the perovskite phase, whereas a (8 × 5 × 2) grid was used for the yellow phase. Both atomic positions and lattice parameters were allowed to fully relax.

## 3. Results

### 3.1. Intrinsic Bulk Properties of the Yellow and Black Phase in CsPbI_3_

**Figure 1:**
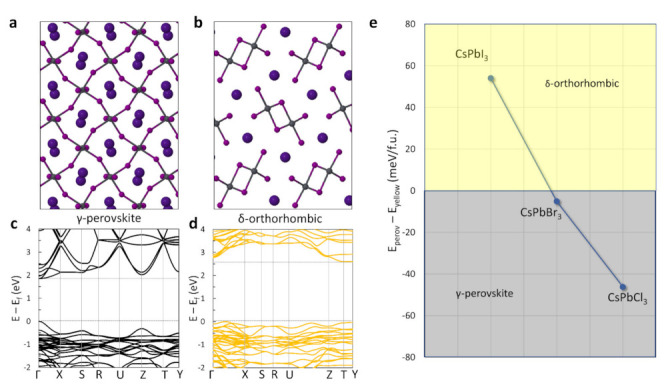
(**a**) Scheme of the γ-CsPbI_3_ perovskite phase seen from the (001) plane; (**b**) Scheme of the orthorhombic δ-CsPbI_3_ yellow phase seen from the (010) plane; (**c**) Band structure of the γ-CsPbI_3_ perovskite phase; (**d**) Band structure of the orthorhombic δ-CsPbI_3_ yellow phase; (**e**) Difference in the calculated ground state energy (at 0 K) between the γ and the δ phases of CsPbX_3_ perovskites (where X = I, Br, Cl).

**Figure 2:**
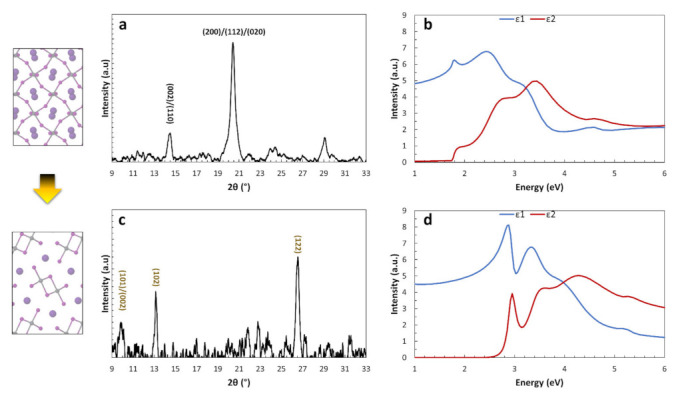
Structural and optical properties of the γ-CsPbI_3_ perovskite phase and its transition towards the δ-CsPbI_3_ yellow phase at room temperature: (**a**) XRD spectrum of the γ-CsPbI_3_ phase; (**b**) Real (ε_1_) and imaginary (ε_2_) parts of the dielectric function for the γ-CsPbI_3_ phase; (**c**) XRD spectrum of the δ-CsPbI_3_ yellow phase; (**d**) Real (ε_1_) and imaginary (ε_2_) parts of the dielectric function for the δ-CsPbI_3_ yellow phase.

CsPbI_3_ can be crystallized in four polymorphic phases that are distinguished by different structures and symmetries. The α, β, and γ phases crystallize in a perovskite structure with either cubic (α), tetragonal (β), or orthorhombic (γ) symmetry. Out of the three, the γ phase has the smallest ground-state energy and it is therefore the most favorable perovskite phase for bulk crystals at relatively low temperatures. At high temperatures instead (>300 °C), the cubic α-perovskite spontaneously forms due to a combined beneficial effect of lattice vibrations/disorder and thermal expansion that lowers the free energy of this perovskite system with respect to all other phases. The orthorhombic (δ) structure instead is a non-perovskite crystal with a formation energy that is even lower than the γ perovskite phase [31]. Figure 1a,b shows the structural characteristics of the γ and the δ phases, respectively, which, under certain conditions, can be both experimentally observed at room temperature. The γ-perovskite is characterized by the typical [PbI_6_]^4−^ octahedra in perovskite crystals, forming Pb-I cages that accommodate the Cs^+^ monovalent cations. The δ phase is instead characterized by quasi-one-dimensional [PbI_3_]^−^ chains that are surrounded by Cs^+^ cations, making the structure highly anisotropic towards the three crystallographic directions. The band structures of the two phases by means of the DFT (Figure 1c,d) provide evidence of why the former is suitable for optoelectronic devices whereas the second lacks photoactivity. For the γ phase, a direct band gap with a value of ~1.75 eV is calculated (Figure 1c), which makes this system ideal for tandem solar cells and potentially useful in other optoelectronic devices. The δ phase instead shows an indirect wide band gap of ~2.6 eV (Figure 1d) that makes it inappropriate for light harvesting applications. We note here that bandgaps within the DFT-PBE approach for these two systems approximate well the experimental values (see Figure 2b,d).

The DFT can also give a clear first-order evaluation of the energetic equilibrium between the two phases (Figure 1e), where the ground-state energy of the yellow phase is lower by 54 meV per formula unit (corresponding to 216 meV per unit cell) from that of the γ black phase. This important difference is practically translated in the spontaneous phase transformation of the γ-perovskite towards the δ phase at room temperature. It is interesting to note here that this preference exists only for the iodide perovskite, whereas for bromide and chloride structures the ground-state energy of the perovskite γ-phase prevails over the δ phase. Indeed, experimentally, CsPbBr_3_ and CsPbCl_3_ perovskites are stable and the manifestation of a δ-orthorhombic phase similar to CsPbI_3_ is not observed [19,32].

In this respect, the main structural and optical characteristics of the two CsPbI_3_ phases can be observed in Figure 2, by means of X-ray diffraction and spectroscopic ellipsometry. In both cases a perovskite polycrystalline layer was obtained through solution processing of a mixture of PbI_2_, CsI, and EuCl_3_, where the presence of the Eu cation guarantees the formation of a relatively stable low-temperature perovskite phase at 80 °C [21], which persists after cooling down to room temperature for several days in a nitrogen ambient. We note here that similar results are obtained if EuI_2_ is used in the solution instead of EuCl_3_, as the europium cation acquires the +2 oxidation state within the perovskite, regardless of its initial state within the solution (see Ref. [26] for a comprehensive discussion on this issue). The XRD data (Figure 2a) show the characteristics of this low-temperature perovskite phase, with diagnostic peaks in the angular range 2θ~14–22°. A deconvolution of the peak at ~20.3° indicates the presence of three separate peak components that correspond to the (200), (112), and (020) planes of the orthorhombic γ-CsPbI_3_. It is therefore important to note that the prevailing perovskite phase at room temperature retains orthorhombic characteristics, in agreement with the lower ground-state energy of this structure as compared to the α and β polymorphs. By fitting the entire pattern with a γ-orthorhombic CsPbI_3_ lattice, the lattice parameters were extracted by means of a standard Rietveld refinement: a = 8.632 Å, b = 8.927 Å and c = 12.327 Å (Pbnm). This dark-colored perovskite phase unavoidably turns into the yellow δ phase at room temperature, either by a gradual transformation within a nitrogen environment (from days to weeks), or rapidly, if exposed to humid air. The main XRD characteristics of the γ-to-δ transformation (Figure 2c) are the peaks at low angles and in particular the diagnostic peaks at 2θ~9.9° and ~13.1°. The Rietveld refinement in this case reveals the following lattice parameters: a = 10.421 Å, b = 4.604 Å, c = 17.873 Å (Pnma). By calculating the respective unit cell volume, it becomes clear that a γ-to-δ transformation induces a volume contraction in the material.

Figure 2b,d shows the real (ε_1_) and imaginary (ε_2_) parts of the dielectric function for the previously discussed phases of CsPbI_3_ measured by spectroscopic ellipsometry. Optically, only the dark perovskite phase has a useful bandgap for devices and applications (~1.77 eV), whereas the yellow crystal is a wide band gap semiconductor (~2.9 eV), in good agreement with our theoretical calculations. Further optical differences appear in the determination of the critical points of the complex dielectric function and on the absorption capacity of the two phases at different photon energies.

By defining the basic structural and optical characteristics of the dark and yellow CsPbI_3_ phases at room temperature, a spontaneous question that arises is in regard to the physical features and quantities that could favor the preservation of the former under device-operating conditions. Interesting insights may come up from the microstructural investigation of the synthesized CsPbI_3_ films through high-resolution scanning transmission electron microscopy, or by theoretical calculations.

### 3.2. Role of Spatial Confinement in the Preservation of the Perovskite Phase of CsPbI_3_

**Figure 3:**
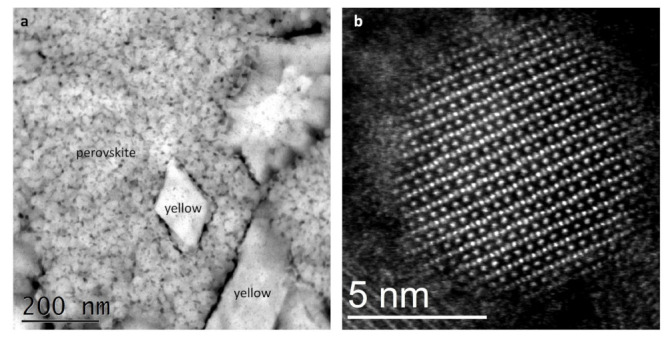
(**a**) STEM image of a γ-CsPbI_3_ perovskite thin film with δ-CsPbI_3_ yellow phase inclusions; (**b**) High-resolution STEM image of a γ-CsPbI_3_ nanograin within a δ-CsPbI_3_ thin film.

To gain an atomic-scale understanding of the structural characteristics of our CsPbI_3_ films, CsPbI_3_ samples were prepared for STEM analysis directly on a C-coated Cu-grid to avoid specific preparation procedures that could alter the material properties. Figure 3a shows a microstructural arrangement of the low-temperature γ-perovskite phase, which is characterized by a continuous network of nanometer-sized grains (~5–20 nm diameter) that are identified from their typical perovskite diffraction pattern, along with inclusions of larger grains that correspond to the yellow phase. The perovskite nanograins are surrounded by a material with amorphous characteristics, which acts as an interconnecting network and renders the entire film compact [26]. The co-presence of both perovskite and yellow grains in the same area of this sample is indicative of an early transition stage and allows for a first consideration on the stability of the perovskite phase, which appears to depend on the small size of the perovskite crystals. Indeed, an analysis of all available samples by STEM showed that larger grains have diffraction patterns that undoubtably unveil the characteristic peaks of the yellow δ-phase. The presence of perovskite nanograins agrees with literature studies that have examined the microstructural arrangement of CsPbI_3_ films starting from different growth methods [24,33]. Indeed, it has been theoretically argued that the surface energy for many low-index Miller planes is significantly lower for the perovskite phase as compared to the yellow orthorhombic structure [24]. Therefore, it appears that a viable route for the stabilization of the perovskite phase at room temperature is by increasing the surface-to-volume ratio of CsPbI_3_ grains, i.e., by confining CsPbI_3_ in nanostructures. This is most probably one of the key roles of europium or other additives during the formation of the CsPbI_3_ perovskite at temperatures that are lower than 300 °C, above which a spontaneous transition to the α-phase takes place.

**Figure 4:**
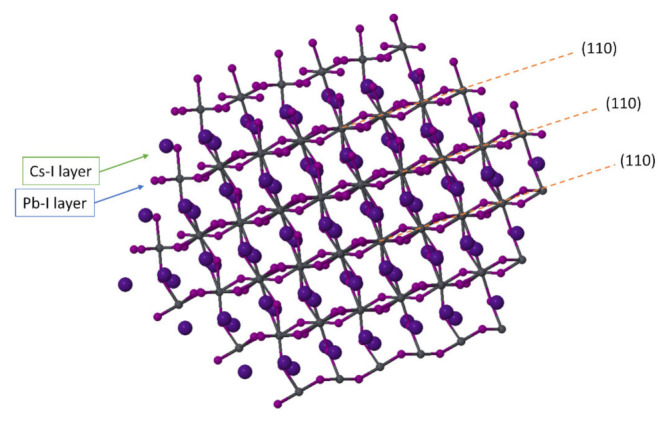
Scheme of a CsPbI_3_ nanograin from the [1–11] zone axis showing the (110) crystallographic planes and the two different types of inorganic layers (Pb-I and Cs-I).

Figure 3b shows an atomic-resolution STEM image of a perovskite nanograin with a diameter of ~9 nm within a thin film that has almost globally transited into the δ-phase. The persistence of this perovskite nanostructure within the yellow layer further supports the role of spatial confinement for the preservation of the perovskite phase and indicates that the transformation from γ to δ may occur through a gradual merging of the initially small grains into bigger structures, where the surface-to-volume ratio decreases. The atomic-scale details of this image are the following: the (1 1 0) perovskite planes are clearly visible from the [1–11] zone axis direction, whereas surfaces seem to be surrounded by an amorphous-like shell. By performing a fine-scale analysis of the atomic structure, it emerges that the [PbI_6_]^4−^ octahedra are tilted (resulting in a zigzag-like configuration for iodine atoms), as expected for the γ-orthorhombic polymorph of CsPbI_3_ (see also Figure 4, where a scheme of a CsPbI_3_ nanograin in the orthorhombic phase is shown). It is important to note here that tilting aids in the stabilization of the CsPbI_3_ perovskite phase, as our theoretical calculations show that the non-tilted α-polymorph has a ground-state energy that is higher than the γ-polymorph by ~124 meV per formula unit. Consequently, the stabilization of the α-phase at high temperatures should largely depend solely on vibrational and volumetric features. 

### 3.3. Role of Electron Concentration and Volume Expansion in the Stabilization of the Perovskite Phase of CsPbI_3_

The high surface-to-volume ratio is an important feature that can shift the energetic equilibrium towards the perovskite phase of CsPbI_3_ at room temperature. However, the formation of thin films that are principally composed of nanograins raises questions on the transport properties of photogenerated carriers in the respective devices, as the presence of a nonuniform lattice could limit the carrier diffusion length and increase the electron-hole recombination rate. In this sense, it is important to investigate alternatives beyond the effect of spatial confinement that could lead to the stabilization of the perovskite phase in crystal grains of bigger dimensions. Within this context, ab initio calculations can be important for exploring some fundamental features of the γ and the δ phases with respect to charge and volume.

**Figure 5:**
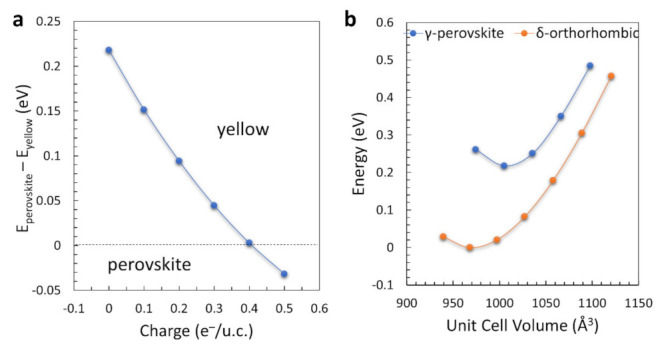
(**a**) Difference in the calculated ground-state energy per unit cell between the γ and the δ phases of CsPbI_3_ as a function of electron doping; (**b**) Relative ground-state energies of γ-CsPbI_3_ and δ-CsPbI_3_ upon volume expansion and contraction. The reference energy at 0 eV has been set to the minimum energy of the δ phase.

Figure 5a shows the energetic competition (at 0 K) between the γ and the δ phase upon an increase of the electron concentration in the material. The uncharged δ-structure has a ground-state energy that is lower by ~216 meV per unit cell with respect to the γ- structure. Upon electron doping, the energy difference tends to diminish and for an electron concentration of ~0.4 e^−^ per unit cell the γ perovskite becomes more stable than the yellow δ-phase. Electron doping could be ideally engineered by partially substituting one or both cationic species of CsPbI_3_ with elements that have a higher oxidation number with respect to Cs^+^ and Pb^2+^, while maintaining a proper coordination with the iodine atoms of the octahedral framework. This practically means that a viable way for the stabilization of the perovskite phase could be the partial substitution of monovalent Cs^+^ cations with divalent cations that can maintain the octahedral perovskite arrangement, or similarly by the substitution of Pb^2+^ with trivalent cations. The possibility of an n-doped CsPbI_3_ material could also open novel possibilities for the engineering of p-n heterojunctions in perovskite solar cell architectures. However, we note that a full stabilization of the CsPbI_3_ perovskite would require a very high level of doping, which could be solely achieved with the formation of stable and uniform perovskite alloys. The latter is one of the most challenging issues in the processing of metal-halide perovskites.

In addition to electron doping, another feature that can create a more favorable energetic framework for the preservation of the perovskite phase is the volume expansion of the material. Figure 5b shows the energetic diagram of the γ and δ phases as a function of the unit-cell volume. For each calculation, only the volume of the structures was fixed, whereas both lattice parameters and atoms were freely allowed to relax. The equilibrium volume of the δ-structure is lower than the one of the γ-perovskite, in agreement with the experimental XRD measurements. By increasing the volume of the lattice (e.g., through artificial strain or thermal expansion at high temperatures) this energetic difference becomes lower. However, even for a lattice expansion as high as 5% with respect to the equilibrium value, the yellow phase remains more stable, indicating that volume expansion can reduce the energetic difference between the two phases, but not stabilize by itself the perovskite structure.

## 4. Discussion

Considering a critical analysis of the available experimental and theoretical data, the main physical aspects that could stabilize the CsPbI_3_ material at room temperature are the spatial confinement in nanocrystalline structures and the level of electron doping in the material. Both features could be practically achieved through the control of the growth process, e.g., by inserting additives in the precursors that can accelerate the perovskite formation at relatively low temperatures though the increase of perovskite nucleation centers during solution processing, while concomitantly substituting the cationic species of the material with atoms that have a higher oxidation number. However, even if the resulting perovskite films are encapsulated in a quasi-inert ambient like nitrogen, a further obstacle that could compromise the medium to long-term stability (i.e., the stability after weeks or months) is the intrinsic capacity of lattice reconstruction in lead-iodide perovskite systems [34], which strongly depends on the weakness of the Pb-I bonds. The latter could accelerate the kinetic merging of the perovskite nanograins (coalescence) when a stable inter-grain blocking layer is absent, resulting in a reduction of the surface-to-volume ratio and a consequent transformation of the films towards the yellow polymorph. Moreover, another characteristic that is closely related to the weak lead-halogen ionic bonding is the self-healing capacity of lead-halide perovskites [35], which should tend to diminish the tolerance for non-intrinsic species over time. Within this context, a primary objective for the long-term stabilization of CsPbI_3_, in addition to the prevalence of the perovskite phase at room temperature, should be the structural reinforcement of the inorganic network. This can be achieved by the partial substitution of iodine with, e.g., bromine [36,37] (considering though the possible implications for the band gap of the material in the related CsPbI_x_Br_3-x_ alloys), the addition of rubidium [38], or by engineering novel processes that could mitigate the facile Pb-I bond breaking and the subsequent iodide kinetics (which should also contribute to ionic currents during device operation).

## 5. Conclusions

We studied the physical aspects that could shift the balance in the energetic equilibrium between the perovskite and the yellow phase of CsPbI_3_, leading to a stabilization of the former at room temperature, by means of theoretical and experimental techniques. We found that two important features that could assist in the stabilization of the perovskite structure are the spatial confinement of CsPbI_3_ nanocrystals and the electron doping of the material. Both features could be engineered during solution processing, e.g., by introducing additives that accelerate the perovskite formation kinetics at relatively low temperatures while concomitantly offering cationic species that can substitute the intrinsic atoms of the material, increasing the electron concentration. We expect further developments on the formation of stable and long-lasting perovskite alloys in the forthcoming years that could mitigate the long-term deterioration of the perovskite structure. 

## Data Availability

The data presented in this study are available on request from the corresponding author.
